# Imagining one experience to be another

**DOI:** 10.1007/s11229-021-03406-y

**Published:** 2021-11-01

**Authors:** Bence Nanay

**Affiliations:** 1 Centre for Philosophical Psychology, University of Antwerp, D 413, Grote Kauwenberg 18, 2000 Antwerp, Belgium; 2 Cambridge, UK

## Abstract

I can imagine a banana to be a phone receiver. I can also imagine the flapping of my arms to be flying. So it is possible to imagine one thing to be another—at least for some types of ‘things’. I will argue that although it is possible to imagine an object to be another object and it is also possible to imagine an activity to be a different activity, one cannot imagine one’s present sensory experience to be a different sensory experience with different qualitative character. This claim will have some important consequences beyond the philosophy of imagination, for example, for some accounts of depiction.

## Imagining X to be Y

Can we imagine X to be Y? For some Xs and Ys, the answer is clearly yes. The standard example of pretense play involves imagining a banana to be a telephone. Another go-to example in the pretense literature is imagining the flapping of your arms to be flying. So for some categories of X and Y, we can indeed imagine X to be Y. But is this the case for all categories of X and Y?

Note that my question is not about how different X and Y can be for us to be able to imagine X to be Y. Imagining a banana to be a phone is possible because they have roughly similar shape (this was even more so when this example was initially used and when phone receivers did have a banana-like shape). One might wonder how similar X and Y need to be, for example, whether it is possible to imagine a banana to be a railway station (seems possible, given the right circumstances), a piece of paper (ditto), an A minor chord (maybe less so), the set of irrational numbers (not sure). These may be interesting questions, but these are not the questions this paper is about. This paper is about whether we can imagine one experience to be another.

The banana and the arm flapping examples show that we can indeed imagine X to be Y as long as X and Y are both objects or if they are both actions. We can imagine one object to be another (there may be some vagueness about what counts as an object in this context, but I put this question aside). We can also imagine one action to be another (again, there is some vagueness here, it is not entirely clear whether and to what extent the arm flapping example can be generalized to mental actions, for example).

The third family of widespread examples of imagining X to be Y is imagining one’s experience to be a different experience (by ‘experience’ I mean ‘sensory experience’ throughout this paper). The aim of this paper is to argue that this is not a feasible imaginative project.

Thus far, I just used the term ‘imagination’ as if it were a unitary concept. But it is not. Imagining comes in many forms. Probably the most commonly drawn distinction between different imaginative episodes is between sensory and propositional imagination. Imagining seeing, hearing, smelling, etc. something are forms of sensory imagination. More generally, as Paul Noordhof says, “the distinctive feature of [sensory] imagining is that a condition of its success is to recreate the sensory experience of the thing imagined” (Noordhof, 2008, p. 337). Propositional imagination is imagining that such and such is the case—imagining that x is F. Imagining seeing the Eiffel Tower from across the river is an example of sensory imagination. Imagining that Paris is the capital of Italy is propositional imagination.

One important question in the philosophy of imagination is about how exactly to draw the line between these two forms of imagination. Another, related, question is which one has anything to do with mental imagery [and by mental imagery I mean perceptual processing not triggered directly by sensory input, see Pearson et al. (2015), Nanay (2018a, 2022)]. We can have mental imagery without imagination (for example when we have an involuntary flashback or an earworm). But how about the other way round? Can we have imagination without mental imagery? In other words, does imagination necessarily involve the exercise of mental imagery (Kind, 2001, Van Leeuwen, 2016, Langland-Hassan, 2020)? The answer depends on what kind of imagination the question is about.

There is broad agreement that sensory imagination, for example, imagining seeing the Eiffel Tower from across the river, does necessarily involve mental imagery (in this case, visual imagery). But there is no agreement about whether mental imagery is necessarily involved in propositional imagination, for example, imagining that Paris is the capital of Italy.

This distinction applies to all the cases of imagining X to be Y I have considered above. It is trivially true that we can imagine for two different objects, X and Y, that X = Y. Propositional imagination of this kind is cheap. That is not the kind of imaginative project that examples that dominate the pretense literature, like imagining a banana to be a phone receiver are about. So the question in that context is whether one can have a sensory imaginary episode of imagining X to be Y. Ditto for imagining action X to be action Y. And ditto for imagining experience X to be experience Y.

Consider the following example: A glass of milk has gone slightly off. While drinking it, however, I try to imagine drinking a glass of fresh milk. It seems that examples of this kind are as widespread as imagining one object to be another or imagining one action to be another. Nonetheless, I will argue that one cannot sensorily imagine one’s actual sensory experience to be a qualitatively different experience (in the same sense modality—I will often drop this qualification to keep things simple, but see Sect. 2 below on why this qualification matters).

It is, of course, very much possible to have some kind of propositional imaginative episode about the identity of two experiences—imagining that the sour milk experience is fresh milk experience. But this is very clearly not what we are trying to achieve in examples of this kind. I assume throughout the paper that the real question about imagining one experience to be another is whether such *sensory* (not propositional) imaginative project is possible.

To drive home just how different sensory and propositional imagination is in this context, it is important to notice that imagining that experience E1 is experience E2 is symmetrical, whereas imagining experience E1 to be experience E2 is not [this is an old point from Williams (1973)]. Imagining that experience E1 is experience E2 is the same imaginative episode as imagining that experience E2 is experience E1. But imagining experience E1 to be E2 is not the same as imagining experience E2 to be E1. Imagining the taste of the sour milk to be fresh is not the same as imagining the taste of the fresh milk to be sour. Everything I say in this paper applies to sensory imagination (and not to propositional imagination).

I have contrasted imagining experience E1 to be E2 with the propositional attitude of imagining that experience E1 is E2. The latter is a feasible imaginative project (most such projects involving propositional imagination are (bracketing examples of imaginative resistance). The former, as I will argue, is not.

But imagining experience E1 to be E2 also needs to be contrasted with another form of imaginative episode, one where E1 and E2 have the same sensory phenomenology. If E1 and E2 have the same sensory phenomenology, then imagining one to be the other is not an instance of sensory imagination, but rather, of propositional imagination. I have just one sensory experience and I propositionally imagine that this experience is a different experience. For those who think that the sensory experience of the fake Vermeer is the same as the sensory experience of the real Vermeer [Danto (1981), p. 1, but see Wollheim (1993), Nanay (2015) for the problems with this assumption], if I am looking at a fake Vermeer painting and I imagine it to be a real Vermeer, this is an imaginative episode that does not involve any form of imagining any experiences. It is another example of propositional imagination: the only thing that is imagined is something non-experiental: that the work was painted by Vermeer. When I talk about imagining experience E1 to be E2 in what follows, I will assume that E1 and E2 do not have the same sensory phenomenology.

Final clarification: I made the contrast between imagining one object to be another and one action to be another on the one hand and imagining one experience to be another on the other much too sharp. In reality, many instances of imagining one object to be another is accompanied by (or maybe even substantially entail) imagining having a certain sensory experience. In pretence cases like imagining a banana to be a phone receiver, we often imagining experiencing the phone receiver. Similar point applies in the case of imagining one action to be another, but as here it is motor imagery that is likely to be involved, I set these cases aside for simplicity.

The structure of the paper is the following: I give two arguments for the claim that one cannot imagine one’s actual sensory experience to be a qualitatively different experience in Sect. 2—an a priori and an empirical one—, and consider some objections in Sect. 3 before outlining some consequences of it for Kendall Walton’s account of depiction in Sect. 4.

## The argument

Imagining that X is Y is a propositional attitude. But imagining X to be Y is an experience. If both X and Y are themselves experiences, then we need to talk about three different experiences and the relation between them. Let’s call the overall imaginative experience E0, the actual experience we imagine to be something else E1 and the experience we imagine E1 to be E2. To put it simply, the overall experience E0 is imagining E1 to be E2.

The first thing to note is that when imagining one’s experience E1 to be E2, both E1 and E2 are part of the content of my overall experience, E0. Take imagining object O1 to be object O2 as an analogy. When I imagine object O1 to be object O2, both object O1 and object O2 need to show up in the content of this imaginative episode—otherwise it would not amount to imagining one thing *to be* another. And the same is true of experiences: imagining one experience to be a different experience implies that the original experience (E1) and the experience you imagine it to be (E2) both show up in the content of the overall imagining episode.

First, take E1. It may happen that I have an experience and that *causes* me to imagine having another experience. Say, I am very thirsty and this experience prompts me to imagine having the experience of a sip of fresh milk. This imaginary project is undoubtedly feasible, but it is not an instance of imagining one experience *to be another*. If I imagine one experience to be another, then it is my present experience that I imagine to be the imagined experience. In other words, the original experience is part of the content of the overall imaginative episode. E1 is part of E0’s content.

Second, take E2. Again, it may happen that you have an experience, and you propositionally imagine that this experience (E1) is E2. This imaginative episode does not necessarily entail having the experience E2. But, as we have seen, it is a very different imaginative episode from imagining E1 to be E2. And assuming that imagining E1 to be E2 is sensory imagination, in order for it to count as imagining something *to be E2*, experience E2 needs to figure in the content of this overall imaginative project (otherwise the imaginative episode would just be something like imagining E1 to be different).

Let’s go back to the sour milk example. While drinking the sour milk, I am trying to imagine drinking a glass of fresh milk. There are two possibilities. If I succeed, the original bad taste is gone, and I only experience the taste of fresh milk. Importantly, the original experience (of the bad taste) is not part of this overall imaginative episode. Even if it was the experience of bad taste that occasioned the imagining process in the first place, it is certainly not part of the content of the imagining it occasions. In this case, E1 (the experience of sour milk) is not part of E0.

If, on the other hand, I do not succeed, that is, if the milk still tastes terrible, then I did not manage to imagine my actual experience to be of a glass of fresh milk: I still experience the terrible taste, I do not experience the taste of fresh milk. I do not manage to imagine my experience *to be some other experience*. The imaginative episode in question just did not happen. In this case, E2 (the experience of fresh milk) is not part of E0.

In short, either the original experience remains or it is entirely replaced by the new imagined experience. Either only E1 but no E2 or E2 but no E1. Neither of these cases constitutes imagining my actual experience to be of something else.

Imagining an experience to be another is an experientially demanding task. If I do indeed succeed in imagining my own experience to be different, this must give rise to a different experience. And in order to imagine *my own actual experience* to be different, my own actual experience also needs to be part of the overall imaginative episode. In short, imagining one experience to be another entails having two phenomenally different sensory experiences at the same time, in the same sense modality.^1^ This seems, intuitively, to be an impossible task, but setting intuitions aside, it is also empirically implausible.

So far, I have appealed to mere intuitions with the milk example (which is quite an extreme example inasmuch as the taste of sour milk and the taste of fresh milk could be thought to be mutually exclusive experiences, which may raise some issues about the generalizability of the example). But there are empirical reasons why one can’t imagine one experience to be another. I will focus on vision for the exposition of this point, but similar considerations apply in the case of other sense modalities as well.

When we see something, our retinal image is transmitted to the retinotopic early cortical areas, first the primary visual cortex, then the secondary visual cortex, and then to a number of still retinotopic areas that are specialized for color, movement, etc. This processing can then have further consequences (for action execution or belief formation). These early cortical areas are retinotopic in the sense that they are (mostly) organized in a way that is homomorphic with the retinal image. To simplify a bit, if I am looking at a simple triangular display, a triangle shape (or something very much like it) can be traced on my retina and a triangle shape (or something very much like it) can also be traced in my primary visual cortex. In short, seeing a triangle implies having a triangle-shape activation in one’s primary visual cortex (and secondary visual cortex, etc.—I will focus on the primary visual cortex for simplicity). To clarify, there is much more involved in having a visual experience than early cortical activation, but early cortical activation is necessary for having a visual experience.

The last paragraph was about the early cortical involvement of perception. But the early cortical areas are also involved in mental imagery. More precisely, in the last couple of decades, there have been a series of empirical findings about the neuroscience of mental imagery and we now know that the early cortical areas are activated in a content-specific manner when one has mental imagery [see, e.g., Kosslyn et al. (2006), Bergmann et al. (2016) and Dijkstra et al. (2019) for summaries]. So when you have mental imagery of a triangle, there will be a triangle-shaped activation in your primary visual cortex (O’Craven & Kanwisher, 2000, Kosslyn et al., 1995). There is a debate concerning whether the involvement of the primary visual cortex is strictly necessary for mental imagery (Bridge et al., 2012), but even if it is not, there is plenty of evidence that the involvement of early cortical areas (that is, not only the primary visual cortex, but the secondary visual cortex and V4) is indeed necessary for mental imagery, see, for example, Kaas et al., (2010). To sum up, it is uncontroversial that activation in the early sensory cortices is necessary for mental imagery and this also holds for the non-visual sense modalities [see Nanay (2018a) for a summary].

With these results in hand, let’s return to the question of imagining one experience to be another. E1, the original experience, is a perceptual experience. So it involves early cortical activation that would correspond to the content of E1. E2 is the imagined experience, which brings with it the mental imagery that involves early cortical activation that would correspond to the content of E2. In short, imagining E1 to be E2 would need to use the early cortical areas twice over, once for E1 and once for E2. But this is not the way the early cortical areas work.

One helpful metaphor used by neuroscientists to describe early vision is that of an ‘active blackboard’ (Bullier, 2001, 2004; Girard et al., 2001; Sterzer et al., 2006). The general idea is that the early visual cortices (and especially the primary visual cortex) function as a blackboard. Various processes can write on this blackboard. Sensory—that is, retinal—stimulation automatically leaves traces on this blackboard, and does so in a retinotopic manner. To simplify a bit, what is on the retina is copied onto the blackboard. But there are other processes that can draw on this blackboard. Mental imagery is the umbrella term for these other processes. Mental imagery tends to influence early vision in a top-down manner—the drawing is done by mechanisms further up in visual processing (Dentico et al., 2014; Mechelli et al., 2004).

This metaphor helps us to appreciate the crucial point about early visual areas: again, to simplify a bit, there cannot be two different patterns at the same time in the same retinotopic region of the primary visual cortex (the same point applies to other parts of early cortical processing as well). So it is either the original experience (E1) that leaves a bottom-up activated trace or the imagined experience (E2) that leaves a top-down activated trace.

The imaginative episode of imagining one experience to be another would need to have both E1 and E2 as its constituents, at the same time. But there are neuroscientific reasons why this can’t be so—we can’t have E1 and E2 at the same time, because we have only one primary visual cortex.

To sum up, the structure of my argument was the following. Imagining one’s present experience to be another is supposed to constitute an experience that is different both from the original experience that we imagine to be different and from the experience we imagine our original experience to be. Imagining experience E1 to be experience E2 constitutes experience E0, which, if this imaginative project is feasible, should be different from both experience E1 and experience E2. But, I aimed to show that, for neuroscientific reasons, experience E0 either collapses into being experience E1, in which case the imaginative project just fails to materialize, or it collapses into being experience E2, in which case one does not experience *one’s original experience* to be another.

## Objections

A couple of possible objections against my argument need to be addressed. First, to return to the original example of drinking milk, the two experiences, the bad and the good taste, may, of course oscillate: I experience the taste of fresh milk for a split second and then the taste of the sour milk for a split second and so on. So even if we can’t have E1 and E2 present as part of E0 simultaneously, we can have E1 and E2 oscillating—would this be enough for the proponents of the feasibility of imagining one experience to be another? While oscillating between two different experiences might be feasible in terms of the neuroscience of the early cortical areas, this is not imagining one experience to be another. This is just having two different experiences change back and forth.

Second, why couldn’t we experience some aspects of the original foul taste and some aspects of the imagined fresh taste—a mixture of the two? Mixed cases like this might be better illustrated with a visual example, say, of looking at a red triangle (E1) and imagining the experience of this red triangle (E1) to be an experience of a yellow square (E2). My argument in this case would amount to saying that both a yellow square (corresponding to E2) and a red triangle (corresponding to E1) can’t show up in the primary visual cortex at the same time. And the objection to this would be that we experience a mixture of E1 and E2—some kind of orange weird-shaped object—a transposition of my actual and the imagined experiences.

There are some interesting examples for this kind of transposition of images in different contexts. In the famous Perky experiments, subjects, who were asked to visualize objects with their eyes open while staring at a white wall, failed to notice when hardly visible images of the visualized objects were projected on this wall (Perky, 1910). In a variant of this experiment, the projected images were different from the ones the agents were asked to visualize and the result was that they ended up visualizing a mixture of the object that was projected on the wall and the object that they were asked to visualize. For example, they were asked to visualize the skyline of New York City while, unbeknownst to them, they were gazing at an image of a red tomato. The result was that they reported visualizing New York City at sunset (see Segal, 1972). Importantly, in this case, the agents were not imagining their experience of the red tomato to be their experience of New York City, and they could not even do so, because they did not know that they were gazing at an image of a red tomato. So even though the transposition of actual and imagined scenes does happen in some cases, this is not what happens when we imagine our actual experience to be something else.

Nonetheless, one version of the worry remains. Wouldn’t it be possible that while the experience E0 is not a mixture of E1 and E2, but imagining E1 to be E2 changes the early cortical activation in a way that it would be a mixture of the typical early cortical correlation corresponding to E1 and the typical early cortical correlation corresponding to E2. The problem with this proposal is that when I imagine the red triangle to be a yellow square, in order for both of these experiences to show up in my overall phenomenology, my primary visual cortex would need to encode both a red triangular shape and a yellow square shape. A catch-all orange weird shape will not give rise to either of these experiences, let alone the experience of imagining one to be the other.

## Consequences: depiction

The claim that we can’t imagine one experience to be another has some far-reaching consequences beyond the philosophy of imagination. In the last section of this paper, I argue that if it is true that we can’t imagine one experience to be another, then Kendall Walton’s account of depiction is highly problematic because he takes the experience of pictures to be an instance of imagining one experience (of the picture surface) to be another (of the depicted scene) (Walton, 1991, p. 404).

One of the most influential recent account of depiction—the question about what makes pictures pictures—is that those objects are pictures that are such that we (are supposed to) go through a certain kind of experience when looking at them. But what experience are we supposed to go through when we are looking at a picture? Ernst Gombrich claims that what characterizes this experience is that our attention alternates between the two-dimensional surface and the three-dimensional represented object when looking at pictures (Gombrich, 1960). Richard Wollheim argued that the experience we are supposed to go through when looking at pictures is a twofold one: we are simultaneously aware of the picture surface and the represented object [Wollheim (1980, 1987, 1998), Nanay (2004, 2005, 2010), for other experiential accounts of depiction, see Lopes (1996), Hopkins (1998)].

Not everyone endorses this ‘experiential’ account of what makes pictures pictures (see Goodman, 1968, Kulvicki, 2006, Peacocke, 1987). But even if one is persuaded by an account of the ontology of pictures that makes no reference to the experience of pictures, it is still a crucial question what makes the experience of a picture of an apple different from the experience of an actual apple. The question about what is distinctive about the experience of pictures is a crucial one not only in philosophy of perception but also in aesthetics.

A particularly well-known account of the experience of pictures was given by Kendall Walton, who claimed that the surface of the picture serves as a prop for what he calls ‘a visual game of make-believe’ (Walton, 1990, esp. pp. 11–68. See also Walton, 1973, 2002a, b, Maynard, 1994, 2012, Carroll, 1995, esp. 97–98). Thus, Walton’s proposal is that experiencing pictures amounts to engaging in ‘a visual game of make-believe’. Walton’s account has become quite influential and although it is often dismissed, it is done so without any thorough argument [for some exceptions, see Wollheim (2002), Nanay (2004), Stock (2008)]. It has also often been misinterpreted or oversimplified. My aim is to apply the argument about imagining one experience to be another in this context to show that Walton’s account of visual games of make-believe posits an imaginative project that is not, in fact, feasible.

In a ‘visual game of make-believe’, on looking at the picture, we imagine our experience of the picture to be of the represented object [Walton (1991), p. 404, see also Walton (2002a, b)]. The question is what this imagining process amounts to. This ‘visual game of make-believe’ is clearly an instance of imagining X to be Y. But what are X and Y? We have seen that it is possible to imagine one object to be another object, but the ‘visual game of make-believe’ is not imagining the picture surface to be the depicted scene. As Walton repeatedly emphasizes, the imaginative episode involves that “a *perception* of the pictorial surface imagined to be a *perception* of […] whatever is depicted” (Walton, 2002a, b, p. 33, my emphasis). This way of thinking about picture perception is the clearest where Walton differentiates visual and other, non-visual, games of make-believe. Walton insists that looking at a picture constitutes a *perceptual* game of make-believe (Walton, 1990, pp. 293–296. esp. p. 296).

We have also seen that it is possible to imagine one action to be another action, but the visual game of make-believe is not an imaginative episode of this kind either. One could insist that when Walton describes the visual game of make-believe as “a *perception* of the pictorial surface imagined to be a *perception* of […] whatever is depicted” (Walton, 2002a, b, p. 33, my emphasis), what is meant by perception is not an experience, but an action (a mental action) (cf. Stock, 2008). But the most straightforward visual actions—like eye movements—don’t seem to be different in the case of looking at a surface and looking at the depicted scene. Thus, imagining one to be the other would not be imagining one action to be a different action—it would be imagining one action to be the very same action, but directed at a different object. But if it is only the object that the action is directed at that is different, then this imaginative episode boils down to imagining one object to be a different object (which we have considered above).

The most charitable way of interpreting Walton’s claim would construe picture perception as imagining one sensory experience to be another sensory experience. And he is quite clear that this is exactly what he means by ‘visual games of make-believe’:

In all of these cases, not only is the actual object of a person’s perceptual experience in fact different from what she imagines it to be and not only does she know this to be so, it is likely that the actual intentional content of her experience is different from what she imagines *it* to be, i.e., the “original experience retains its content” even as she imagines it to have a different content (Walton, 2002a, b, p. 32).

And here is an even more explicit statement, which makes it clear that for him the experience of picture perception involves both the original experience we imagine to be different (E1) and the experience we imagine E1 to be (E2):

The spectator imagines seeing a mill, and also imagines her actual perceiving of the picture to be her perceiving of a mill. These elements are integrated into a single experience which is at once imaginative and genuinely visual. (Walton, 1991, p. 425)

This discussion makes it clear that for Walton the perceptual game of make believe necessarily involves having a perceptual experience of the picture surface (E1) and an imagined experience of the depicted scene (E2), which are part of a third experience of imagining E1 to be E2 (E0). And this whole imaginative project is an instance of sensory imagination as it involves the exercise of mental imagery, an assumption that is especially clear when Walton distinguishes the perceptual game of make believe of picture perception and the kind of game of make-believe we engage in when reading novels (Walton, 1990, pp. 293–294). If I read *L’Éducation Sentimentale*, I (may) form a mental imagery of Madame Arnoux, but I do not imagine *my sensory experience of the pages* to be my experience of her. If I look at a painting of Madame Matisse, on the other hand, I do imagine *my sensory experience of the painting* to be my sensory experience of Madame Matisse. For Walton, both games of make believe involve mental imagery, but the latter involves imagining one experience (our actual one) to be another one.

Further, it is important to emphasize that the imaginative episode Walton talks about is not visualizing. If I am visualizing a chair, my actual experience does not play any role in my imagining the experience of a chair. This is not what Walton means by the visual game of make-believe. He made this clear when answering one of Richard Wollheim’s objections to his account [Walton (1991). See also Walton (2002a, b), esp. p. 33 and Walton (1990), pp. 300–301]. Wollheim interpreted Waltons’ notion of imagining seeing Y as visualising Y: as an imagining process whereby the actual perceptual content plays no role—we could do it even with our eyes shut (Wollheim, 1991, esp. p. 404). In his response, Walton makes it clear that what he means by imagining seeing Y is not visualising, but imagining *one’s actual seeing X* to be one’s seeing Y.

Thus, according to Walton, what constitutes the experience we are supposed to go through when looking at pictures is imagining our actual sensory experience of the canvas to be our experience of the depicted object: imagining one sensory experience to be another.

I argued in the last section that this is not a feasible imaginative project. When I succeed in imagining my seeing the surface (E1) to be my seeing the depicted object (E2), I am experiencing what I would experience if I were seeing the depicted object (E2). My actual experience of seeing the surface (E1) does not play any role in this ‘game of make-believe’. Thus, I do not imagine *my seeing the surface* to be my seeing the depicted object. If, on the other hand, I do not succeed in imagining my seeing the surface (E1) to be my seeing the depicted object (E2), the game of make-believe just does not happen: again, I do not imagine my seeing the surface *to be my seeing the depicted object*. Neither of these is imagining one sensory experience to be another.

This argument is structurally similar to a somewhat sketchy point Richard Wollheim makes in response to Walton’s theory. Wollheim says:

My difficulty […] is how to understand […] imagining one perceptual experience to be another. For if we succeed, in what way does the original experience retain its content? For, what is left of the experience of seeing the surface when I successfully imagine it to be some other experience? However, if I do continue to see the surface, or this experience retains its content, how have I succeeded in imagining it, the experience, to be an experience of seeing a [depicted object]? (Wollheim, 1998, pp. 224–225).

Walton’s response to this argument (Walton, 2002a, b, see also Walton, 2008) is a series of examples that are supposed to show that it is possible to imagine one experience to be another. The general line of reasoning seems to be that there are lots of cases, other than picture perception, where imagining experience E1 to be experience E2 constitutes an experience E0 that is different from both E1 and E2. I will argue that in none of these alleged examples does imagining sensory experience E1 to be experience E2 constitute an experience E0 that is different from both E1 and E2. Thus we have no reason to believe that we are capable of imaginative episodes of this kind.

His examples are the following (Walton, 2002a, b, p. 32):

- Imagining my actual experience of listening to a recording of Glenn Gould’s performance to be my experience of listening to him live,
- Imagining my actual experience of listening to the flutist to be my experience of listening to Papageno’s crude instrument,
- Imagining (unconsciously) my actual experience of interacting with my boss to be my experience of interacting with my father, and finally and most surprisingly,
- In Hitchcock’s *Vertigo*, Scottie imagines his actual experience of Judy, whom he just dressed up in the clothes of Madeleine, to be his experience of Madeleine.

In all these cases, Walton’s point is that the actual experience and the imagined experience are different: they have different content and the agent knows this. Hence, as in all these cases (a)–(d), one imagines one experience to be a different experience, it is clearly possible to imagine one’s experience to be another experience: hence, the same is possible in the case of imagining our experience of the surface to be of the depicted object.

But does he show that we imagine our actual experience to be another experience in the examples (a)–(d)? Is it clear that in any of these cases our imaginative project of imagining our actual experience to be a different experience gives rise to a third experience that is different from both? I don’t think so. Example (d) is especially telling: Scottie does not really imagine his experience of Judy to be his experience of Madeleine: he may have a belief that the person he is looking at is Judy, but he dresses her up as Madeleine precisely in order to have a Madeleine-experience, that is, a Judy-free Madeleine-experience. Judy triggers the Madeleine-experience, but we have no reason to suppose that Judy is part of the content of his imaginative episode. In fact, if Judy were to show up in his imagined experience, this would take away from what Scottie is trying to achieve. Scottie’s imaginative project is a clear and quasi-pathological case of self-suggestion.

I am not sure that example (c) is relevant here at all, as Walton explicitly says that imagining my actual experience of interacting with my boss to be the experience of interacting with my father is an unconscious mental episode. As a result, it makes little sense to ask how the phenomenal characters of the two experiences relate to one another as experience E2 is supposed to be unconscious, hence, it is not an experience. In short, this example does not show that we can imagine our actual experience to be another.

How about the other two examples? It is not clear whether there is a phenomenal difference between the actual and the imagined sensory experiences in (a) and (b). But regardless of whether we take these two experiences to have the same sensory phenomenology or not, the examples are not convincing. There are two options: the sensory phenomenology of these two experiences are either the same or different. I’ll take these two options in turn.

First suppose that there is no difference in sensory phenomenology. Suppose that the objects of these experiences are different, but in ideal conditions, listening to Glenn Gould’s recording and listening to him live could be indistinguishable in terms of sensory phenomenology. The same goes for Papageno’s flute-play. Thus, the sensory phenomenal character of experience E1 and the sensory phenomenal character of experience E2 are the same. They sound exactly the same.

As we have seen in Sect. 1, assuming that the experience of a fake Vermeer and the experience of a real Vermeer are the same (an assumption many would question), it may be a feasible imaginative project to imagine one’s experience of a fake Vermeer to be a real Vermeer, because the sensory phenomenology of these two experiences are the same, hence, the imaginative episode involved is propositional imagination. But, crucially, even if this is what is going on in the Gould and the Papageno examples, this is clearly not what happens in the case of depiction. When we look at pictures, the experience of the canvas is very different from the experience of the depicted scene, as Walton would be the first to acknowledge: “Hobbema’s Water Mill looks like what it is—a paint-covered stretch of canvas—not at all like a red-roofed water mill” (Walton, 1990, p. 298). So even though this may be a genuine case of imagining A to be B, it does not help any account of depiction.

The second option is that there is a difference in sensory phenomenology between the actual and the imagined experience in examples (a) and (b)—listening to Gould’s recording and listening to him live, are phenomenally different. Say, I’m listening to an old vinyl of the Gould recording, which makes crackling noises. Applying the argument from above, what we get is that there are two options when I imagine this experience to be of listening to Gould live. First, if the crackling noise is not part of my sensory experience (of imagining one to be the other), then the original experience is gone. Second, if the crackling noise is still part of my sensory experience (of imagining one to be the other), then it is unclear that I managed to imagine my actual experience to be of listening to a live performance. Neither of these cases constitutes imagining one sensory experience to be another.

Finally, I need to address a somewhat indirect potential objection. I argued that the imaginative episode Walton posits is not a feasible one because one can’t have two different sensory experiences at the same time. But Richard Wollheim, whom I sided with to oppose Walton, himself argued for an account of pictorial experience that is a twofold experience that is, in some sense simultaneously about the picture surface and the depicted scene. So how is the pictorial experience Wollheim posits any better off than the one Walton posits when it comes to the objection raised in this paper? My response is that it is because Wollheim is very clear that the twofold experience is not two experiences, but one, which has two aspects, the configurational aspect (which is about the picture surface) and the recognitional aspect (which is about the depicted scene). According to Wollheim, we do not have two different experiences when we look at pictures. We only have one, twofold experience (see esp. Wollheim, 1998, but see also Nanay, 2018b). According to Walton, in contrast, if we imagine one experience to be another when we look at pictures, then we do indeed have two different experiences—and I aimed to show that this is an implausible position. In short, nothing I said here should jeopardise the claim that pictorial experience is twofold—understood in a Wollheimian way.

Time to sum up: Although it is possible to imagine one object to be another and to imagine one activity to be another, there are empirical reasons to suppose that it is not possible to imagine one’s actual sensory experience to be different. As Walton’s theory of experiencing pictures postulated that we imagine our sensory experience of the surface to be of the depicted object, this theory is deeply problematic for empirical reasons.^2^

## Footnotes

1 Some further qualifications may be required besides ‘at the same time, in the same sense modality’. For example, in the visual sense modality, it seems possible to see a purple patch of color on the left side of one’s visual field, but at the same time, in the same sense modality, imagine a yellow patch of color in the right side of one’s visual field. The empirical argument that follows in the main text gives us an argument why this would be so. Thanks to an anonymous referee for raising this issue.

2 This work was supported by the ERC Consolidator grant [726251], the FWF-FWO grant [G0E0218N] and the FWO research grant [G0C7416N]. I wrote an early version of this paper in the early 2000s and I presented it at a number of conferences under the title ‘Imagining seeing and imagining kissing’. I presented it at the National Postgraduate Analytical Philosophy Conference (UK) in 2004 or 2005, at the Eastern APA in 2006, at the Annual ASA in 2006, at the 2007 Depiction workshop in Manchester, also once at UBC maybe in 2006. Special thanks for comments by the audiences and by my assigned commentators at these events, Jennifer Hudin, Achim Spelten, Christopher Bartel and last but not least Kendall Walton whose criticism convinced me to set aside that old paper for good. I’m grateful to Nick Wiltsher for trying to convince me years later to keep it set aside… I also got written comments from Jerrold Levinson, Dom Lopes, John Kulvicki, David Davies, Michael Newell, Rob Hopkins and Robert Van Gulick as well as 3 or 4 referees from this journal. But my greatest debt is to Richard Wollheim whose initial enthusiasm for this paper is the reason I have decided to revise it almost 20 years after having written it.
